# The extracellular matrix protein SNED1 affects self-renewal, proliferation and adipogenic differentiation of human adipose stem/progenitor cells

**DOI:** 10.1080/21623945.2026.2737919

**Published:** 2026-09-27

**Authors:** Sonja Großmann, Petra Waldegger, Tina Deutinger, Werner Zwerschke

**Affiliations:** a Division of Cell Metabolism and Differentiation Research, Research Institute for Biomedical Aging Research, University of Innsbruck, Innsbruck, Austria; b Center for Molecurrlar Biosciences Innsbruck, University of Innsbruck, Innsbruck, Austria; c Department of Plastic, Reconstructive and Aesthetic Surgery, Medical University of Innsbruck, Innsbruck, Austria

**Keywords:** Adipogenesis, extra cellular matrix, SNED1, human adipose stem cells, proliferation

## Abstract

The fate of adipose stem/progenitor cells (ASCs) is influenced by the extracellular matrix (ECM). However, the interaction between ASCs and components of the ECM in human white adipose tissue (WAT) is not precisely understood. The gene Sushi, nidogen and EGF-like domain-containing protein 1 (SNED1) encodes a secreted ECM glycoprotein of unknown function in AT. In the present study, we show that knockdown of the SNED1 expression in ASCs leads to a significantly reduced self-renewal and proliferation capacity; moreover, the adipogenic differentiation potential is reduced. Our data suggest that SNED1 contributes to a functional interaction between ECM and ASCs, which is important for stemness and commitment to proliferation and differentiation in adult adipose stem/progenitor cells.

## Introduction

White adipose tissue (WAT) plays an important part in obesity [1]. It is a loose connective tissue containing aggregates of white adipocytes essential for triglyceride storage and metabolic homoeostasis. According to the current model, adipocytes arise out of an adipose lineage involving multipotent adult stem cells, progenitor populations, and preadipocytes with the capacity for self-renewal, proliferation, and terminal adipogenic differentiation [2,3]. The adipose stem and progenitor cells (ASCs) are important for adipose tissue homoeostasis, regeneration [4–8], and expansion [9–11]. Determining the fate of ASCs involves a complex integration of signal transduction pathways, such as insulin, IGF-1, PDGF, TGF β  and WNT signalling but also cues from the cytoarchitecture of the adipose stem cell niche [12]. Such signals can trigger proliferation by modifying quiescence/cell cycle factors [13] and orchestrating terminal adipogenesis by activation of transcription factors of the CCAAT/enhancer-binding protein (C/EBP) family and peroxisome proliferator-activated receptor *γ* (PPAR *γ*) [14].

A major niche for ASCs in human WAT is the vascular interface surrounding small blood vessels [15–18]. An important structural determinant of the niche is the extracellular matrix (ECM), a three-dimensional (3D) meshwork consisting of various proteins and glycans, forming a scaffold surrounding and supporting cells [19,20]. Its composition can affect diverse cell parameters, including migration, proliferation, and differentiation. Pioneering studies have shown that the composition of the ECM is influencing AT cells. 3T3-L1 preadipocytes secrete ECM proteins during adipogenic differentiation [21,22]. Fibronectin can inhibit the differentiation of 3T3‑F442A cells into adipocytes [23]. Moreover, integrin-alpha-5, which interacts with fibronectin in the ECM, can enhance proliferation and attenuate adipocyte differentiation in 3T3-L1 preadipocytes [24]. However, the interaction between ECM and ASCs in WAT is still poorly understood. We identified the ECM protein Sushi, Nidogen, and EGF-like Domain-containing protein 1 (SNED1) in a screening for weight-loss (WL) target genes in human ASCs [25–29]. SNED1, alias Stromal nidogen extracellular matrix protein (Snep), was first described by Leimeister et al., 2004 in mice as a gene expressed during embryo development encoding for a secreted ECM glycoprotein [30]. SNED1 knockout studies in mice suggest a role for SNED1 in morphogenic processes and neonatal survival [31]. In adult humans, SNED1 was described as a promoter of cancer metastasis [32–34]. As precedence exists suggesting that alterations in the ECM can influence AT stem cell functions, and SNED1 is a secreted ECM protein, the aim of this study is to analyse whether SNED1 functionally interacts with the activation of ASCs. By employing a gene silencing approach, this study highlights the ECM protein SNED1 as a novel regulator of ASC self-renewal, proliferation, and adipogenic differentiation.

## Material and methods

### Adipose tissue donors

The human white adipose tissue samples were derived from patients undergoing plastic surgery at the Institute for Plastic, Reconstructive and Aesthetic Surgery of the Medical University Innsbruck, Austria (Table 1). All patients gave their written consent, and the study has been approved by the ethical committee of the Medical University of Innsbruck according to the Declaration of Helsinki (ethic committee board number: AN 3047 257/4.13).

**Table 1.** Clinical anthropometric parameters of sWAT donors.

Donor	Sex	Age	Height (cm)	Weights_tart_ (kg)	BMI_Start_	Weight_Final_ (kg)	BMI_Final_
1	f	28	162	112	43	60	23
2	f	40	–	–		73	N.a.
3	m	21	173	130	43	75	25
4	f	41	165	93	34	63	23
5	f	30	167	135	48	58	21
6	m	31	182	154	46	91	27

Human sWAT samples were obtained from healthy donors undergoing routine abdominoplasty after weight loss at the Department of Plastic, Reconstructive and Aesthetic Surgery at the Medical University of Innsbruck, Austria. f = female, *m* = male, BMI_Start_ = body mass index before weight loss, BMI_Final_ = body mass index after weight loss, Weight_Start_ = weight before weight loss, Weight_Final_ = weight after weight loss, at surgery, n.a. not available.

### Cell culture

ASCs were seeded in DMEM/ F-12 medium with HEPES (4-(2-hydroxyethyl)-1‑piperazineethanesulfonic acid) and L-glutamine containing 33 μM biotin, 17 μM pantothenate, 20 μg/ ml ciprofloxacin and 10% FBS (ASC2 medium). The following day, the medium was changed to proliferation medium consisting of DMEM/F-12 medium with HEPES and L-glutamine containing 33 μM biotin, 17 μM pantothenate, 10 ng/ml EGF, 1 ng/ml FGF, 500 ng/ml insulin and 20 μg/ml ciprofloxacin supplemented with 2.5% FBS, referred to as PM4. Before adipogenesis, cells were starved in DMEM/F-12 medium with HEPES and L-glutamine containing 33 μM biotin, 17 μM pantothenate and 20 μg/ml ciprofloxacin, without FBS (ASC1 medium).

### Isolation of ASCs and SVF of human white adipose tissue

ASCs were isolated under sterile conditions as described previously [13]. Briefly, after surgery the tissue was rinsed with PBS, dissected and about 50 g of tissue was incubated in 150 ml of sterile filtered 200 U/ml collagenase (CLS Type I, Worthington Biochemical Corp., Lakewood, NJ) in 2% w/v BSA in PBS for 60–90 min at 37 °C while stirred at 450 rpm. The solution was sieved to remove resting debris and undigested tissue, followed by centrifugation for 10 min at 200 × g RT temperature, and adipocytes were removed. The pellet containing the SVF was treated with erythrocyte lysis buffer (0.155 M Na_4_Cl, 5,7 mM K_2_HPO_4_, 0.1 mM EDTA, pH 7.3) and filtered through a cell-strainer (pore size 100 μm). After centrifugation, the cells were resuspended in ASC2 medium, filtered through a 35 µm cell strainer and seeded at high density in a 6‑well plate. Medium was changed after 16 h to ASC1. After 6 days in serum-free culture, the cells were reseeded in flasks and cultured until 70% density in PM4 growth medium before harvesting, freezing and storing in nitrogen.

### Induction of adipogenesis

Confluent ASCs were cultivated in ASC1 for 2–4 days before adding differentiation medium consisting of ASC1 medium supplemented with 0.2 µM insulin, 0.5 mM 3-isobutyl-1-methylxanthine (IBMX), 0.25 μM dexamethasone, 2.5% foetal bovine serum and 10 μg/mL transferrin. IBMX was only included for the first 3 days, and the medium was changed every 3 days.

### shRNA mediated knock-down

Two shRNA containing lentiviral vectors (pLKO.1) targeting the SNED1 gene were obtained from Dharmacon (Dharmacon, Lafayette, LA, USA; sh1 (TRC0000056123), sh2 (TRCN0000056127)) to achieve knock-down in human ASCs and a non-targeting shRNA was used as control (scr). The plasmid DNA was isolated using the EndoFree Plasmid Maxi Kit (#12362, Qiagen, Hilden, Germany). HEK293 FT cells were used for production of lentiviral particles by combining the plasmid DNA, psPAX packaging plasmid and pMDG.2 envelope plasmid with Lipofectamine 3000 reagents (Life Technologies #L3000015). Virus particles were stored at −80 °C, and the titre was determined in U2OS cells. For infection, 6–8×10^5^ ASCs were seeded in a 175 cm^2^ flask in ASC2 medium, which was changed to PM4 the next morning, and infected after 6 h with a multiplicity of infection of 4 in 15 ml PM4 including 6 µg/ml Polybrene. Medium was changed to PM4 the next day, followed by selection in PM4 including 2 µg/ml Puromycin for 3 days.

### Proliferation assay

Cells were seeded at 2 × 10^4^ per well in ASC2 in 6‑well plates. The next day (day 0), the medium was changed to PM4 and changed every 2–3 days. The cells were counted with a FACS Canto II (BD Bioscience) at a medium flow rate for 90 s.

### BrdU incorporation assay

BrdU incorporation was assessed by using the 5-Bromo-2‘-deoxy-uridine Labeling and Detection Kit III (Roche, 11,444,611,001) according to the manufacturer’s manual. Briefly, 1600 cells per well were seeded in ASC2 medium in 96‑well plates, including three technical replicates. The next day, the medium was changed to PM4 with or without BrdU (10 µM final concentration). On days 4 and 6, the cells were fixed, incubated with nuclease working solution and stained with anti-BrdU-POD Fab fragments (200 mU/ml final concentration). After incubation with POD-substrate, absorbance was measured at 490 nm.

### Colony formation assay

To assess colony‑forming capacity, 1000 cells per well were seeded in ASC2 medium in 6-well plates. The medium was changed to PM4 medium the following day and refreshed every 2–3 days. At day 14, the cells were fixed with Methanol/Acetone (1:1) for 3 min and stained with crystal violet for 30 min. Colonies of six technical replicates were counted.

### Western blot

For analysis of protein levels, cells were harvested in SDS (sodium dodecyl sulphate) lysis buffer. After sonication, protein concentrations were determined by using the Compat-Able Protein Assay Preparation Reagent Set (Thermo Fisher Scientific, #23215, Vienna, Austria) and the Pierce BCA Protein Assay Kit (Thermo Fisher Scientific, #23227, Vienna, Austria) according to the manufacturer’s instructions. 10–20 μg of total protein per sample were used for separation by SDS-polyacrylamide gel electrophoresis (SDS-PAGE) and transferred onto PVDF membranes. As primary antibodies, anti-FABP4 (#10004944, Cayman) and anti-PPARγ (#23215, Cell Signalling) were used. The secondary antibody was goat anti-rabbit IgG-HRP (#W4011, Promega). Chemiluminescent signals were detected using a ChemiDoc imaging system (BioRad, Feldkirchen, Germany), and membranes were stained with Ponceau S for normalization to total protein. Densitometric analysis was performed using ImageJ v1.51n (NIH, USA) and Image Lab v6.0.1 (Bio-Rad, Feldkirchen, Germany).

### Rt-qPCR

RNA was isolated and purified using the RNeasy Plus Micro Kit (Qiagen, #74034, Hilden, Germany) according to the manufacturer´s instructions. RNA concentration was measured using a NanoDrop spectrophotometer (Thermo Scientific, Vienna, Austria). For cDNA synthesis, 500 ng RNA was reverse transcribed using the First Strand cDNA Synthesis Kit (Thermo, #1622, Vienna, Austria). The cDNA was diluted 1:5 for real-time quantitative PCR (RT-qPCR). RT-qPCR was performed on a QuantStudio^TM^ Flex System (Thermo Fisher Scientific, Vienna, Austria) using AceQ SYBR Green Master Mix (Vazyme, Q111-02, Nanjing, China). All samples were measured in technical triplicates. β-Actin was used as a housekeeping gene for calculation of relative gene expression by the ∆∆C_T_ method. Primer sequences are listed in Table 2.

**Table 2.** Primer sequences.

Target	Sequence	
	forward	reverse
β-Actin	AGA AAA TCT GGC ACC ACA CC	AGA GGC GTA CAG GGA TAG CA
FABP4	TACTGGGCCAGGAATTTGAC	GTGGAAGTGACGCCTTTCAT
Perilipin	GACAACGTGGTGGACACAGT	CTGGTGGGTTGTCGATGTC
PPARγ	ATGGGTGAAACTCTGGGAGA	TGGAATGTCTTCGTAATGTGGA
SNED1	CACTCCGGACTCTACGTGAAC	AGGTGGCATTGAAGTCCAGG

### Triglyceride staining

Triglyceride accumulation in differentiated ASCs was assessed by Oil Red O staining 14 days after induction of adipogenesis. The cells were fixed with 4% paraformaldehyde for 1 h at RT, washed with PBS and stained for 1 h with 0.5% Oil Red O prepared in isopropanol/water (60/40). For quantification, Oil Red O was extracted with isopropanol and extinction was measured at 570 nm, and/or stained cells of at least 6 pictures of each construct were counted. Each condition was assessed in ASC derived from at least three donors in three technical replicates.

### Statistical analysis

GraphPad Prism 5 was used for statistical analysis. Two-way ANOVA with a Bonferroni post-test was used for RT-qPCR analysis and growth curves; otherwise, a paired two-tailed t-test was used. Mean ± SEM is shown in every graph, and the significances are shown as followed: * ≙ *p* < 0.05, ** ≙ *p* < 0.01, *** ≙ *p* < 0.001. Detailed information is provided in each figure legend.

## Results

### SNED1 is influencing proliferation and self-renewal of human ASCs

It has been shown that murine preadipocyte lineages secrete ECM proteins during adipogenic differentiation that can interfere with preadipocyte functions [21–24]. As SNED1 is a secreted ECM protein [30,31], we wanted to assess whether SNED1 influences the proliferation of ASCs. To do this, we knocked down (KD) SNED1 using gene‑specific short-hairpin RNAs (shRNA) in ASCs isolated from human subcutaneous (s)WAT (Figure 1a), and determined the effect on cell proliferation by establishing growth curves and performing BrdU incorporation assays. The SNED1 KD was confirmed at the RNA level with an average reduction of 82% for sh#1 and 50% for sh#2 and resulted in reduced proliferation of ASCs, as indicated by significantly decreased cell numbers (Figure 1b) and BrdU incorporation (Figure 1c). Additionally, self-renewal capacity, assessed by colony‑formation assays showed significantly reduced colony‑forming units in SNED1 KD ASCs by an average reduction of 35% for sh#1 and 75% for sh#2 in cells from three different donors (Figure 1d). Our data suggest that SNED1 contributes to the proper self-renewal and proliferation capacity of human ASCs.

**Figure 1.**
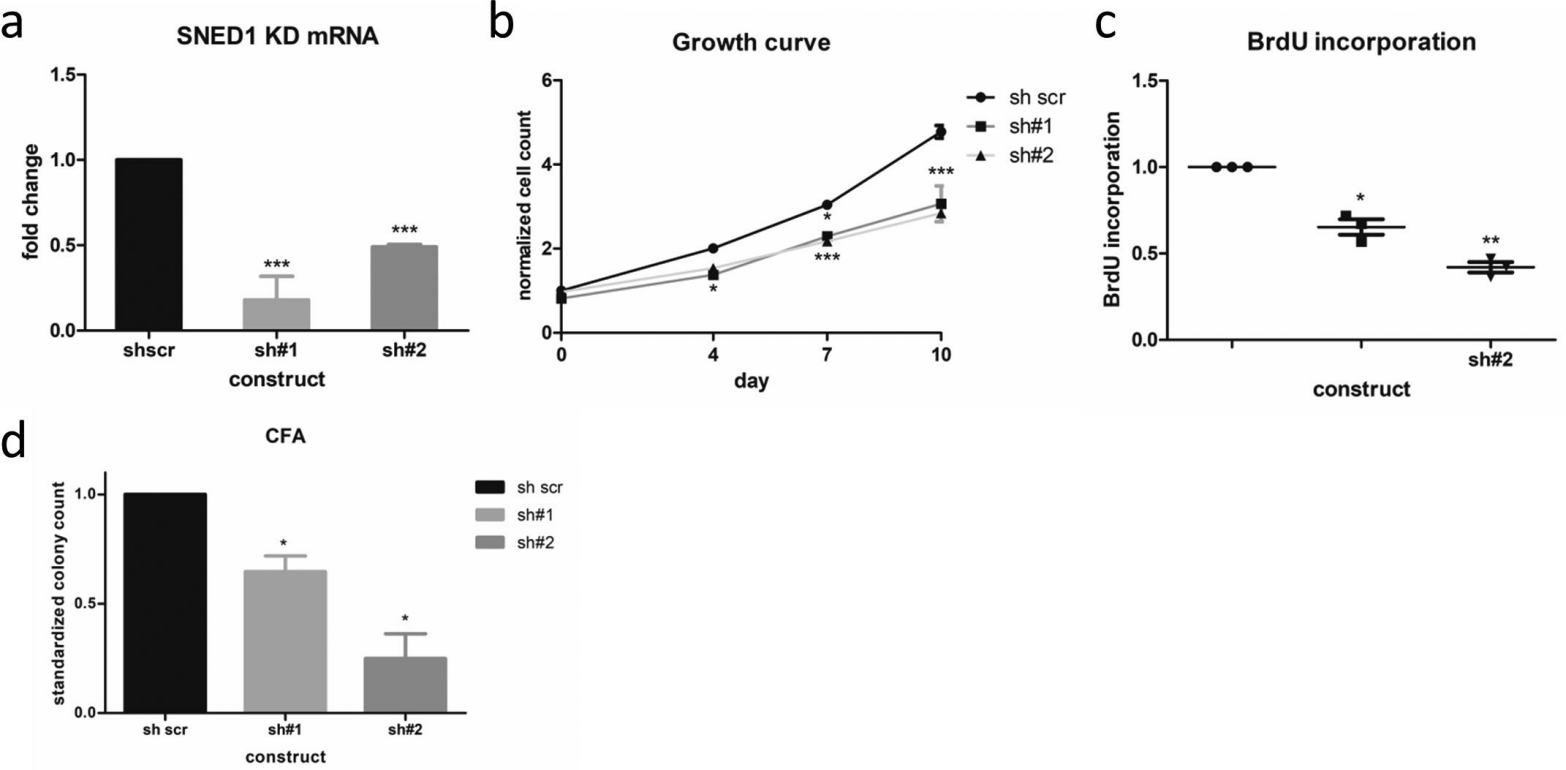
SNED1 knock-down reduces proliferation and self-renewal capacity of ASC.

(a) SNED1 shRNA‑mediated knock-down in ASCs was confirmed by qRT-PCR; sh#1 = anti-SNED1, shRNA1, sh#2 = anti-SNED1 shRNA2, sh scr = control. Data represent the mean of three technical replicates from three different donors. A two‑tailed paired t-test was performed for analysis. Values are Mean ± SEM across donors. (b) Growth curves of SNED1 KD and control ASCs from one representative donor out of three different donors are shown. Mean ± SEM of three technical replicates is shown, and a two-way ANOVA with Bonferroni post-test was performed. (c) BrdU incorporation of SNED1 KDs and control ASCs, *n* = 3 donors; three technical replicates per donor and shRNA were averaged. A paired two-tailed t-test was used for statistics, and values are shown as Mean ± SEM (d) The self-renewal capacity of the SNED1 KDs and control ASCs was measured by Colony Formation Assays (CFAs). Results from *n* = 3 biological replicates (donors) are shown as Mean ± SEM. Data represents standardized colony count of the mean of six technical replicates per donor; the means of each donor were used for analysis with a two-tailed paired t-test. Significance: * ≙ *p* < 0.05, ** ≙ *p* < 0.01, *** ≙ *p* < 0.001.

### SNED1 is influencing adipogenic differentiation of human ASCs

As we could find an increase in SNED1 expression in ASCs of donors after weight-loss (Supplementary Figure S1), we analysed the adipogenic differentiation process in SNED1 KD ASCs. RT-qPCR analysis shows a reduction of the mRNA expression of the adipocyte markers PPARγ, FABP4 and Perilipin in SNED1 KD cells (Figure 2a–c). Western blots show reduced protein levels of PPARγ and FABP4 in SNED1 KD cells (Figure 2d–f). Oil-Red-O staining after 14 days of adipocyte differentiation shows reduced triglyceride content in both SNED1 KD constructs (Figure 2g). The data suggest that SNED1 contributes to regulation of adipogenic differentiation of human ASCs.

**Figure 2.**
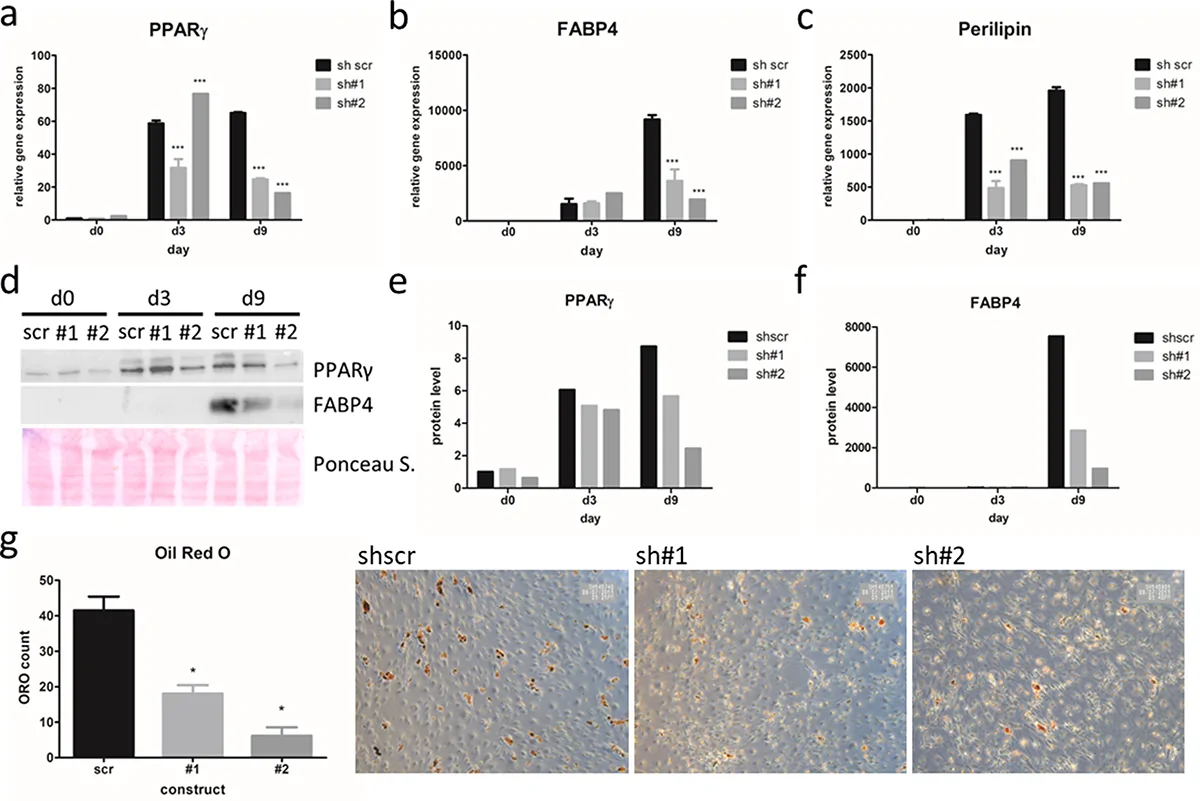
SNED1 knock-down reduces adipogenic differentiation in ASCs.

Adipogenesis was induced in SNED1 KDs and control ASCs. The mRNA expression of (a) PPARγ, (b) FABP4 and (c) Perilipin was assessed at indicated time points by RT-qPCR. Results of one representative donor out of three different donors are shown. For each time point, 3 technical replicates (wells) were measured, shown as Mean ± SEM. Two-way ANOVA with a Bonferroni post-test was used for analysis. (d) Western blots were performed, and densitometric analysis of (e) PPARγ and (f) FABP4 protein levels was normalized to total protein level by Ponceau S staining. Representative results of one out of three donors are shown. (g) Oil-Red-O staining was performed 14 days post-induction of adipogenesis. Left panel: Mean ± SEM of 3 different donors is shown. A two‑tailed paired t-test was performed with the mean of three technical replicates per donor. Right panel: representative pictures of Oil-Red-O staining. Significance: * ≙ *p* < 0.05, ** ≙ *p* < 0.01, *** ≙ *p* < 0.001.

## Discussion

Fibroblast feeder layers support adipogenesis in human ASCs [35] and cultivation of ASCs in a WAT organoid model increases adipogenic differentiation capacity [36]. Moreover, cell-ECM contact [21–24] and cellular confluency [37,38] are requirements for adipogenesis. While these findings suggest that ECM and surrounding niche cells are important for ASC fate decisions, it was shown that the composition and stiffness of the ECM are important for AT remodelling [39]. In fact, it can influence quiescence, proliferation and adipogenic differentiation of human mesenchymal stem cells [40] and plays a role in lineage commitment of these cells [41,42]. In addition, ECM remodelling mediated by ECM structure modifiers such as matrix metalloproteinases and tissue inhibitors of metalloproteinases was shown to regulate adipocyte progenitor commitment and competency [43–47]. Thus, to better understand regulation of ASC fate in AT, it is important to intensify studies addressing the interaction between the ECM and these cells.

SNED1 is a novel ECM protein with roles in the adhesion of breast cancer and neural crest cells and most likely in the proliferation of mouse embryonic fibroblasts [30,48,49]. Until now, the role of SNED1 in AT was unknown. In the present study, we demonstrate that silencing of SNED1 in human ASCs leads to reduced self-renewal, proliferation and adipogenic differentiation capacity. These data suggest that the ECM protein SNED1 plays a part in the regulation of the fate of ASCs by different programmes. We have previously presented data suggesting that ASCs in sWAT predominantly exist in a quiescent state [13]. Since loss-of-function of SNED1 reduces the self-renewal capacity of ASCs, it is conceivable that SNED1 influences the stemness of adult adipose stem cells. Together with the abrogation of proliferation in SNED1 KD ASCs, these data suggest a role for SNED1 in maintaining the quiescent stage or in leaving it and entering the cell cycle. The reduction of proliferation in SNED1 KDs suggests also a role for the ECM protein in amplification and hence lineage commitment of ASCs. This is further underscored by the reduced capacity of SNED1 KD ASCs to undergo terminal differentiation into adipocytes. The data presented in our study provide evidence suggesting a role of the ECM protein SNED1 in regulating the fate of ASCs. This underscores the importance of the ECM for governing quiescence and commitment of ASCs.

Our findings are a prerequisite for future studies aimed at determining to what extent SNED1 contributes to the regulation of the adipose lineage. Cues from the ECM are sensed and transmitted by members of the integrin family and other types of cell surface receptors [20,50]. Integrins have been shown to regulate cellular processes such as proliferation [51], senescence [52], and differentiation [53], and are important for tissue homoeostasis in adult organisms [54]. More recent studies suggest that SNED1 interacts with integrin signalling [48], and integrins have been implicated in the proliferation and differentiation of adipose stem and progenitor cells [24,55]. Thus, a role for a SNED1/Integrin axis in ASC is conceivable; however, further research must show by which mechanisms SNED1 affects ASC.

## Supplementary Material

Supplemental Material

## Data Availability

Data are available within the article or its supplementary material or can be requested from the authors.
